# Acute Inhibition of Selected Membrane-Proximal Mouse T Cell Receptor Signaling by Mitochondrial Antagonists

**DOI:** 10.1371/journal.pone.0007738

**Published:** 2009-11-10

**Authors:** Kwangmi Kim, Lin Wang, Inkyu Hwang

**Affiliations:** Department of Chemistry and Chemical Biology, The Scripps Research Institute, La Jolla, California, United States of America; New York University School of Medicine, United States of America

## Abstract

T cells absorb nanometric membrane vesicles, prepared from plasma membrane of antigen presenting cells, via dual receptor/ligand interactions of T cell receptor (TCR) with cognate peptide/major histocompatibility complex (MHC) plus lymphocyte function-associated antigen 1 (LFA-1) with intercellular adhesion molecule 1. TCR-mediated signaling for LFA-1 activation is also required for the vesicle absorption. Exploiting those findings, we had established a high throughput screening (HTS) platform and screened a library for isolation of small molecules inhibiting the vesicle absorption. Follow-up studies confirmed that treatments (1 hour) with various mitochondrial antagonists, including a class of anti-diabetic drugs (i.e., Metformin and Phenformin), resulted in ubiquitous inhibition of the vesicle absorption without compromising viability of T cells. Further studies revealed that the mitochondrial drug treatments caused impairment of specific membrane-proximal TCR signaling event(s). Thus, activation of Akt and PLC-γ1 and entry of extracellular Ca^2+^ following TCR stimulation were attenuated while polymerization of monomeric actins upon TCR triggering progressed normally after the treatments. Dynamic F-actin rearrangement concurring with the vesicle absorption was also found to be impaired by the drug treatments, implying that the inhibition by the drug treatments of downstream signaling events (and the vesicle absorption) could result from lack of directional relocation of signaling and cell surface molecules. We also assessed the potential application of mitochondrial antagonists as immune modulators by probing effects of the long-term drug treatments (24 hours) on viability of resting primary T cells and cell cycle progression of antigen-stimulated T cells. This study unveils a novel regulatory mechanism for T cell immunity in response to environmental factors having effects on mitochondrial function.

## Introduction

T cell activation, a series of physiological changes leading to clonal expansion and development of effector functions, commences as T cell receptor (TCR) encounters a cognate peptide in the context of major histocompatibility complex (MHC) presented by specialized immune cells called antigen presenting cells (APCs). The interaction of TCR with cognate peptide/MHC complex (pMHC) triggers a host of intracellular signaling cascades leading to cell cycle progression [1]. Despite crucial, TCR/pMHC interaction is generally insufficient for the productive T cell activation, for which accessory (costimulatory) receptor/ligand interactions, typified by CD28/B7-1 and lymphocyte function associated antigen-1 (LFA-1)/intercellular adhesion molecule-1 (ICAM-1), are required [2], [3].

LFA-1, a member of β2 integrin family, plays multiple roles in T cell immunity both as an adhesion and a signaling molecule [4]. Thus, LFA-1/ICAM-1 interaction not only promotes firm and stable T/APC interaction but also triggers signaling cascades for T cell activation. The functional property of LFA-1 is carefully regulated during T cell immunity via transition from low affinity/avidity state to high affinity/avidity state and vise versa. The functionality of LFA-1 is controlled by a signaling mechanism, called “inside-out” signal, elicited by distinct neighboring receptors such as TCR and chemokine receptors [5], [6].

Mitochondria are a powerhouse of cells generating ATP via oxidative phosphorylation/respiratory electron transport system. The rate of ATP production is affected by various environmental factors such as oxygen stress (e.g., hypoxia), nutritional condition and molecules interfering with oxidative phosphorylation [7]. Thus, it is necessary to control the rate of ATP consumption (e.g., anabolic metabolism for cell cycle progression) in response to those environmental changes to preserve the pool of ATP needed for basal cell metabolism. Here, a mechanism for sensing the cellular energy level is indispensible and AMP-activated protein kinase (AMPK) plays the role [8].

Mitochondria also emerge as a center for control over cell signaling. Reactive oxygen species (ROS) (e.g., O_2_^−^ and H_2_O_2_) and reactive nitrogen species (RNS) (e.g., NO) play a role in cell signaling as second messengers [9]. Superoxide (SO), i.e., O2^−^, is produced as a byproduct of oxidative phosphorylation [10]. NO is also produced in mitochondria by mitochondrial NO synthase (mtNOS) [11]. In addition, importance of mitochondria in receptor-mediated extracellular Ca^2+^ entry has been revealed; mitochondria act as an intracellular Ca^2+^ buffer prolonging the Ca^2+^ entry and thereby potentiating downstream signaling cascades [12].

T lymphocytes absorb APC-derived nanometric membrane vesicles, naturally-occurring exosome-like membrane vesicles (eMVs) or artificially prepared plasma membrane-derived membrane vesicles (pMVs), via dual receptor/ligand interactions of TCR/pMHC plus LFA-1/ICAM-1[13], [14]. Studies using 2C TCR transgenic (Tg) T cells along with pMVs prepared from artificial APCs, Drosophila (Dros) cells expressing L^d^ (a mouse class I MHC) plus mouse ICAM-1 and B7-1, also have shown that the vesicle absorption requires intracellular signaling events. The signaling mechanism commences as 2C TCR interacts with cognate pMHCs expressed in the pMVs and is thought to be critical for promoting LFA-1 function [15].

Applying those findings, we had established a cell-based flow cytometric high throughput screening (HTS) platform for isolation of small molecules causing inhibition of 2C T cell absorption of L^d^B7-1ICAM-1 pMVs, which can be exerted by either physical interference of TCR/pMHC or LFA-1/ICAM-1 interaction or disruption of TCR-mediated ‘inside-out’ signaling [15]. As an effort to validate the practicality and effectiveness of the HTS platform, we had carried out a pilot-scale HTS campaign with a commercial library composed of both natural and synthetic molecules whose biological functions are known to differential extents and found that molecules known to antagonize the mitochondrial function (oxidative phosphorylation) inhibited the pMV absorption. In this study, we investigated effects of mitochondrial drug treatments on various membrane proximal TCR signaling events and assessed the potential use of those molecules as immune modulators.

## Results

### Inhibition by Various Mitochondrial Antagonists of 2C T cell Absorption of L^d^B7-1ICAM-1 pMVs

Exploiting the simplicity and statistical accuracy of the assay for 2C T cell absorption of L^d^B7-1ICAM-1 pMVs, we had established a HTS platform for isolation of small molecules interfering with the pMV-absorption and screened a commercial library composed of natural and synthetic molecules whose biological functions are known to differential extents [15].

A intriguing finding from the pilot-scale HTS campaign was that molecules known to antagonize mitochondrial function (i.e., oxidative phosphorylation) were isolated as hits; a group of rotenoids represented by Deguelin (a Complex I inhibitor) [16] and Antimycin (a Complex III inhibitor) [17]. IC_50_s of Deguelin and Antimycin were determined at 300 and 5 nM, respectively, and toxicities of those molecules against 2C T cells were not detected when measured after the assay by propidium iodide exclusion method.

**Figure 1:**
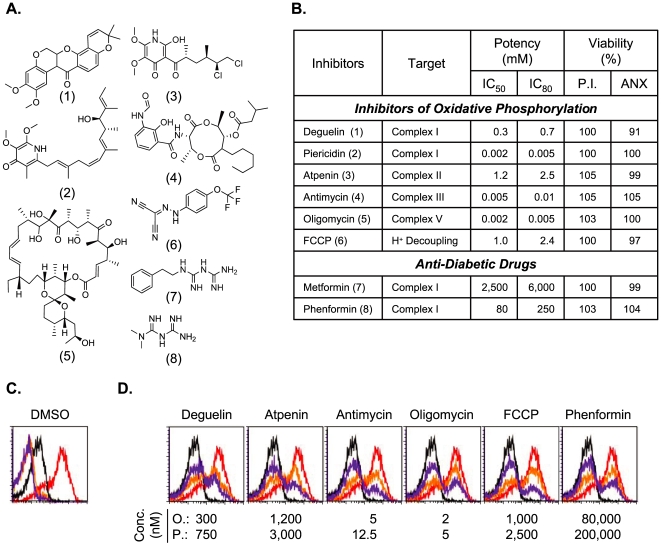
Potencies of various mitochondrial antagonists for inhibition of the pMV-absorption and their toxicities against 2C T cells. (A) Chemical structures of mitochondrial antagonists (1: Deguelin, 2: Piericidn, 3: Atpenin, 4: Antimycin, 5: Oligomycin, 6: FCCP, 7: Phenformin, 8: Metformin) are shown. (B) Assays for absorption of L^d^B7-1ICAM-1 pMVs were performed with 2C T cells pre-treated with titrated concentrations of respective mitochondrial antagonists for 1 hr. IC_50_ and IC_80_ were determined with mean fluorescence intensities (MFIs) of B7-1 staining of 2C T cells. Viabilities of 2C T cells were measured after treatment with respective mitochondrial antagonists at their IC_80_s for 1.5 hrs with propidium iodide (P.I.) and Annexin-V staining, respectively. Shown are viabilities of drug-treated 2C T cells relative to that of DMSO-treated 2C T cells. (C) Histograms represent B7-1 staining of 2C T cells left untreated (purple) or treated with DMSO alone for 1 hr followed by cultured with P1A- (orange) or QL9-loaded L^d^B7-1ICAM-1 pMVs in the presence (black) or absence (red) of anti-LFA-1 mAb. (D) Histograms represent B7-1 staining of 2C T cells pretreated with DMSO alone (black and red) or with respective mitochondrial antagonists at two separate concentrations (Orange: O., Purple: P.) as indicated and cultured for 30 min with QL9-loaded L^d^ B7-1ICAM-1 pMVs in the presence (black) or absence (red, orange and purple) of anti-LFA-1 mAb.

Those results prompted us to examine other mitochondrial antagonists with distinct mechanisms of action; Piericidin (a Complex I inhibitor) [18], Atpenin (a Complex II inhibitor) [19], Oligomycin (a Complex V inhibitor) [20] and FCCP (an H^+^ decoupling agent) [21]. As summarized in Figure 1, all of those compounds strongly inhibited the pMV-absorption without revealing noticeable toxicity against 2C T cells as measured by either propidium iodide exclusion or Annexin V staining. Flow cytometry data showing the pattern of inhibition by the mitochondrial antagonists of the pMV-absorption are presented in Figure 1D.

It is perceived that a class of anti-diabetic drugs, i.e., biguanides such as Metformin and Phenformin, exert their pharmacological activities via interruption of mitochondrial respiratory electron transport chain; likely via inhibition of Complex I [22], [23]. As other mitochondrial antagonists, both Metformin and Phenformin also inhibited the pMV-absorption (Figure 1).

### Metabolic Changes Effected by the Mitochondrial Drug Treatments

As an effort to understand underlying mechanism(s) for the inhibition by the mitochondrial antagonists of the pMV-absorption by 2C T cells, we first examined metabolic changes resulting from the drug treatments. Effects of the mitochondrial drug treatments on cellular energy balance, defined as ratio of ([AMP] + [ADP]) to entire pool of adenine nucleotides ([AMP] + [ADP] + [ATP]), were examined measuring relative concentrations of respective adenine nucleotides using HPLC. (Note: HPLC elution chromatograms of Deguelin-treated samples are presented as examples in Figure S1.)

**Figure 2:**
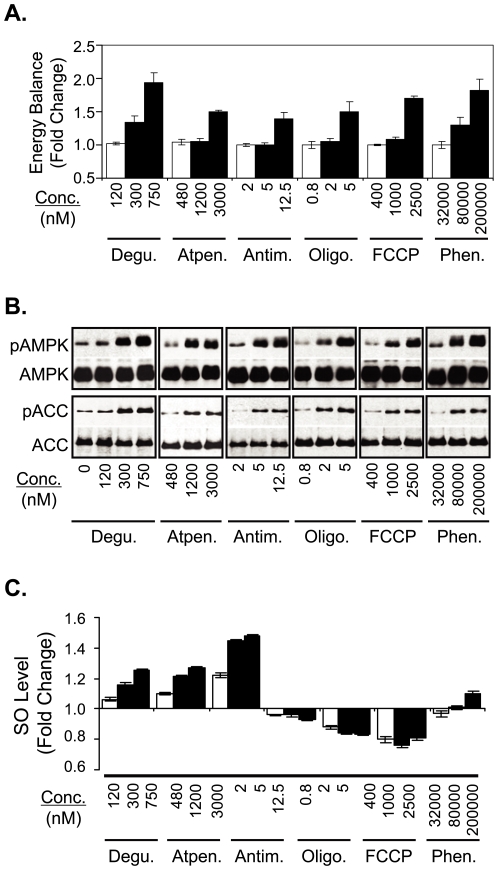
Metabolic changes elicited by mitochondrial drug treatments. (A) Relative concentrations of adenine nucleotides in 2C T cell extracts prepared after treatment with titrated concentrations of respective mitochondrial antagonists (75 min) were measured by HPLC analysis. Energy balance is defined as ratio of ([AMP] + [ADP]) to entire pool of adenine nucleotides, ([AMP] + [ADP] + [ATP]). Energy balances of drug-treated 2C T cells relative to that of DMSO-treated 2C T cells are plotted. Experiments were performed in duplicate. (B) Cell extracts prepared from 2C T cells after treatments with respective mitochondrial antagonists (75 min) were subjected to Western blotting (WB) for phospho-AMPKα1 (Thr^172^) and phospho-ACC (Ser^79^), respectively. WB analysis (re-blot) for AMPKα1 and ACC are also shown as loading controls. (C) Levels of intracellular superoxide (SO) of 2C T cells treated with respective mitochondrial antagonists, relative to that of 2C T cells treated with DMSO alone, are plotted. The SO level was measured by loading the cells with diethylethidium followed by flow cytometric analysis. Experiments in this Figure were repeated at least three times and the representative data are shown.

Treatments (75 minutes) of 2C T cells with the respective mitochondrial antagonists at concentrations near their IC_80_s for the pMV-absorption (Deguelin: 750 nM, Atpenin: 3000 nM, Antimycin: 12.5 nM, Oligomycin: 5 nM, FCCP: 2500 nM, Phenformin: 2000000 nM) ubiquitously increased the cellular energy balance by decreasing the concentration of ATP and increasing concentrations of ADP and AMP. The maximum change was observed when the cells were treated with Deguelin at 750 nM; as a result, ratio of the concentration ATP to that of total adenine nucleotides dropped from 88% to 63% (Figure 2A). When the cells were treated with the respective drugs at concentrations near the IC_50_s for the pMV-absorption (Deguelin: 300 nM, Atpenin: 1200 nM, Antimycin: 5 nM, Oligomycin: 2 nM, FCCP: 1000 nM, Phenformin: 80000 nM), however, changes in the cellular energy balance were hardly detected, except for Deguelin and Phenformin treatments. Even in those cases, the ratio of the concentration of ATP to that of total adenine nucleotides dropped only by about 4% and increase in the concentration of AMP was not detected (Figure 2A).

The mitochondrial drug treatments resulted in activation of AMPKα1; AMPKα1 is an isotype of AMPK predominantly expressed in T cells [24]. Thus, phosphorylation of AMPKα1 at threonine 172, which is critical for its enzymatic activity [25], was ubiquitously detected in dose-dependent manner after treatments (75 minutes) of 2C T cells with the respective mitochondrial antagonists. The phosphorylation of AMPKα1 was accompanied by the phosphorylation of acetyl-CoA carboxylase (ACC), an enzyme involved in lipid biosynthesis and one of the best known cellular targets of AMPK (Figure 2B) [8].

The mitochondrial drug treatments had effects on intracellular SO level as well (Figure 2C). Notably, however, the drug effects showed disparate and appeared to be related with the target of the molecules; thus, treatments with Complex I and II inhibitors (Deguelin and Atpenin, respectively) resulted in a noticeable increase in the intracellular SO level while treatments with a Complex V inhibitor (Oligomycin) and a H^+^ decoupling agent (FCCP) resulted in a slight decrease in the SO level. The effects of Antimycin and Phenformin treatments were neutral.

### Effects of the Mitochondrial Drug Treatments on Activation of Akt and PLC-γ1 and Entry of Extracellular Ca^2+^

Various signaling events proceed rapidly upon contact of 2C T cells with QL9-loaded L^d^B7-1ICAM-1 pMVs, which include phosphorylation of Akt and phopsholipase C-γ1 (PLC-γ1) and entry of extracellular Ca^2+^ [15], [26]. While interaction of 2C TCR with L^d^/QL9 complex is indispensable for those signaling events, costimulatory receptor/ligand interactions (i.e., CD28/B7-1 and LFA-1/ICAM-1) also play prominent roles [27], [28]. (Note: QL9 peptide forms a complex with L^d^ and the L^d^/QL9 complex interacts with 2C TCR with high affinity [29].).

**Figure 3:**
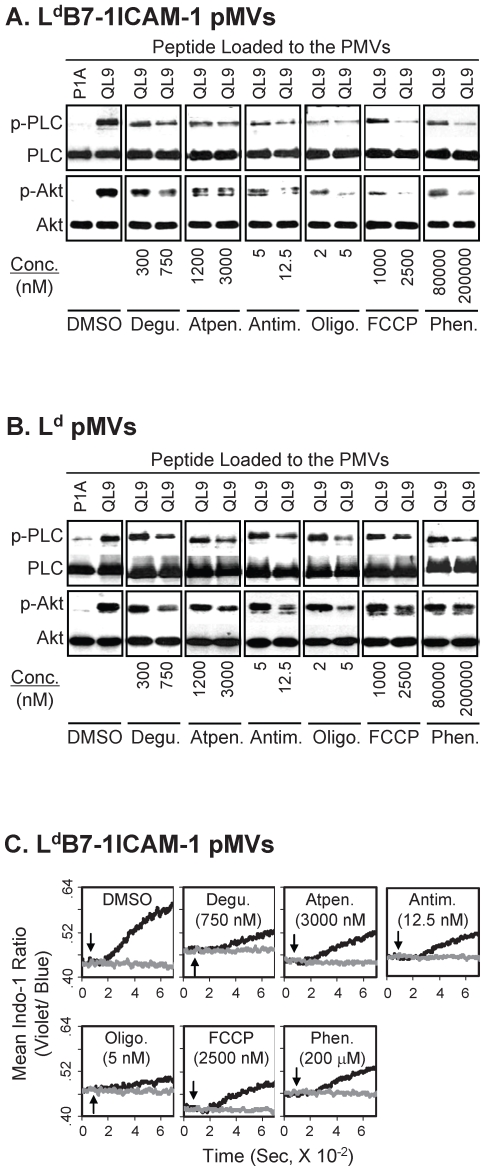
Inhibition by the mitochondrial drug treatments of activation of PLC-γ1 and Akt and Ca^2+^ entry by L^d^B7-1ICAM-1 pMVs. (A and B) Cell extracts prepared from 2C T cells, pre-treated with titrated concentrations of respective mitochondrial antagonists (60 min) and then cultured for 15 min with peptide-loaded L^d^B7-1ICAM-1 (A) or L^d^ (B) pMVs, were subjected to WB analysis for phospho-PLC-γ1 (Tyr^783^) and phosph-Akt (Thr^308^), respectively. WB analysis (re-blot) for PLC-γ1 and pan-Akt are also shown as loading controls. Refer to Figure S2 for relative intensities of bands of WB shown in B. (C) Ca^2+^ assays were performed with 2C T cells loaded with Indo-1. 2C T cells treated with the respective mitochondrial antagonists for 60 min were mixed with P1A (grey) or QL9 (black) peptide-loaded L^d^B7-1ICAM-1 pMVs (marked by arrows) and analyzed for changes in cytosolic [Ca^2+^] for 12 min.

The mitochondrial drug treatments (60 minutes) resulted in ubiquitous inhibition of phosphorylation of both PLC-γ1 and Akt occurring upon culture of 2C T cells with QL9-loaded L^d^B7-1ICAM-1 pMVs. The inhibition was obvious even after treatments of 2C T cells with the respective mitochondrial antagonists at concentrations near the IC_50_s (Figure 3A). The same treatments also inhibited the phosphorylation of Akt and PLC-γ1 in 2C T cells cultured with pMVs expressing L^d^ alone in a dose-dependent manner (Figure 3B), assuring their effects on TCR signaling.

Entry of extracellular Ca^2+^ was also attenuated by those treatments (Figure 3C). (Note: In reflection of the role of mitochondria as an intracellular Ca^2+^ store, the mitochondrial drug treatments themselves, without stimulation by the pMVs, resulted in changes in cytosolic Ca^2+^ concentration to a certain extent, shown by altered background fluorescence intensities of Indo-1-loaded 2C T cells.)

### Lack of Inhibition by the Mitochondrial Antagonists of F-Actin Polymerization

F-actin content of 2C T cells increases quickly upon culture with QL9-loaded L^d^B7-1ICAM-1 pMVs. Different from phosphorylation of Akt and PLC-γ1 and Ca^2+^ entry, interaction of 2C TCR with L^d^/QL9 complex is necessary and sufficient for near maximum level of F-actin polymerization and the costimulatory receptor/ligand interactions play minimal roles [15].

**Figure 4:**
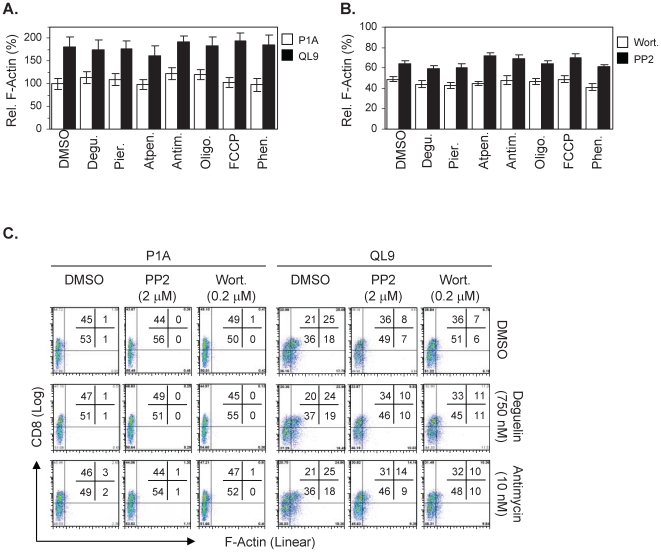
Lack of inhibition by the mitochondrial drug treatments of F-actin polymerization by L^d^B7-1ICAM-1 pMVs. (A) 2C T cells pre-treated (60 min) with the respective mitochondrial antagonists (or DMSO) at concentrations near the IC_80_s for the pMV absorption (Deguelin; 750 nM, Atpenin; 3000 nM, Antimycin; 12.5 nM, FCCP; 2,500 nM, Phenformin; 200,000 nM) were cultured for 15 min with peptide-loaded L^d^B7-1ICAM-1 pMVs followed by F-actin staining and flow cytometric analysis. F-actin contents of 2C T cells, relative to that of 2C T ells treated with DMSO and cultured with the P1A-loaded pMVs, are plotted. (B) 2C T cells, pre-treated (60 min) with the respective mitochondrial antagonists (or DMSO) at concentration as in (A) along with either Wortmannin (0.2 µM) or PP2 (2 µM), were cultured for 15 min with the QL9-loaded pMVs followed by staining for F-actin. F-actin contents of 2C T cells co-treated with the respective mitochondrial antagonists plus Wortmannin (or PP2) are compared with that of 2C T cells treated with the respective mitochondrial antagonists alone. (C) Raw data obtained after treatments with DMSO alone, Deguelin and Antimycin, respectively, along with PP2 or Wortmannin are shown as representatives.

In striking contrast to the signaling events described above, the mitochondrial drug treatments, even at concentrations around the IC_80_s for the pMV-absorption, exerted little effects on the F-actin polymerization. Thus, 2C T cells pre-treated (60 minutes) with the respective mitochondrial antagonists showed the comparable levels of F-actin content, when measured by flow cytometry after 15 minute culture with the QL9-loaded pMVs, as 2C T cells treated with DMSO alone (Figure 4A and C).

In conformation of the importance of protein tyrosine kinase(s) (PTK) and phosphoinositide-3-kinase (PI3K) in membrane-proximal TCR signaling leading to the F-actin polymerization [30], both PP2 (a PTK inhibitor) and Wortmannin (a PI3K inhibitor) treatments strongly attenuated the F-actin polymerization (Figure 4B and C). Likewise, co-treatments of 2C T cells with PP2 (or Wotmannin) plus the respective mitochondrial antagonists also resulted in inhibition of the F-actin polymerization, supporting that the mitochondrial drug treatments had little effect on TCR-mediated PTK(s) and PI3K activation for the F-actin polymerization.

### Effects of the Mitochondrial Drug Treatments on Dynamic Rearrangement of F-Actin

**Figure 5:**
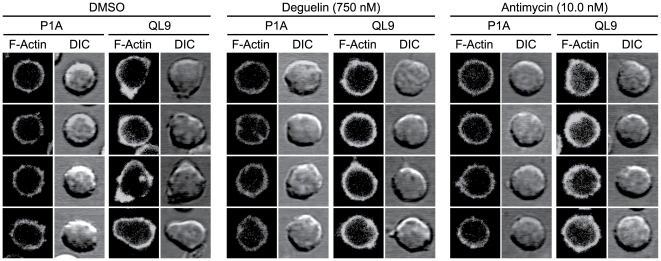
Impairment of dynamic rearrangement of F-actin by the mitochondrial drug treatments. 2C T cells, pre-treated for 60 min with the respective mitochondrial antagonists (or DMSO alone) at concentrations as in Figure 4 were cultured for 20 min with the peptide-loaded L^d^B7-1ICAM-1 pMVs on poly-L-lysine-coated coverslips followed by F-actin staining. Confocal microscopic images of fluorescence F-actin staining and differential microscopic contrast (DIC) of 2C T cells treated with DMSO, Deguelin and Antimycin, respectively, are shown as representative examples.

We also examined 2C T cells stained for F-actin under confocal microscope. As reported, 2C T cells cultured with QL9-loaded L^d^B7-1ICAM-1 pMVs showed enhanced cortical F-actin staining compared with control 2C T cells cultured with the P1A-loaded pMVs [15]. Consistent with results from flow cytometric analysis, 2C T cells, pre-treated with the respective mitochondrial drugs and then cultured with the QL9-loaded pMVs, also showed considerably higher levels of cortical F-actin staining than 2C T cells pre-treated with the same mitochondrial antagonist but cultured with the P1A-loaded pMVs (Figure 5). (Note: Figure 5 shows microscopic images of 2C T cells treated with DMSO alone, Deguelin and Antimycin, respectively, as representative examples. 2C T cells treated with other mitochondrial antagonists also showed the similar pattern.).

Of interest, however, 2C T cells treated with the respective mitochondrial antagonists showed relatively even distribution of cortical F-actin along the cell surface compared with 2C T cells treated with DMSO alone. In addition, membrane protrusion and morphological changes, apparent in DMSO-treated 2C T cells, were not evident in mitochondrial drug-treated T cells, indicating that dynamic rearrangement of F-actin following F-actin polymerization was impaired by the mitochondrial drug treatments (Figure 5).

### Effects of Long-Term Mitochondrial Drug Treatments on Viability of Resting T Cells

TCR signaling is crucial not only for activation/proliferation of T cells encountering cognate foreign antigens but also for survival of resting T cells [31]; TCR signaling for the latter is instigated by interaction of TCR with specific self peptide-MHC complexes. The data that the mitochondrial drug treatments acutely inhibited specific membrane-proximal TCR signaling events prompted us to examine effects of the long-term drug treatments on viability and activation/proliferation of T cells.

**Figure 6:**
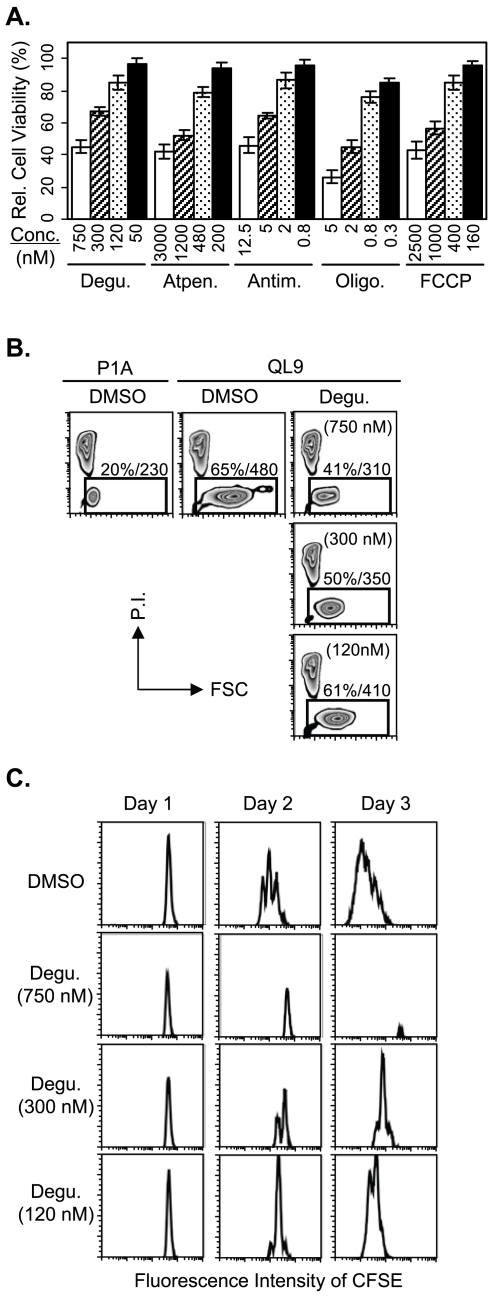
Effects of long-term mitochondrial drug treatments on viability and activation of T cells. (A) Ex vivo purified whole lymph node cells (B6) were treated for 24 hrs with titrated concentrations of the respective mitochondrial antagonists. Viabilities of the T cells in the culture were determined based on P.I. exclusion and compared with that of the T cells treated with DMSO alone (1% V/V). (B) Shown are P.I. staining and sizes (forward scatter, FSC) of 2C T cells analyzed after culture for 24 hrs with the peptide-loaded L^d^B7-1ICAM-1 pMVs in the presence of DMSO alone or titrated concentrations of Deguelin as indicated. Percentages of live (gated P.I.-low) cells and their mean FSC are denoted inside plots; percentage of live cells/mean FSC. (C) Purified CFSE-labeled CD8^+^ 2C T cells were cultured with P1A or QL9-loaded L^d^B7-1ICAM-1 pMVs in the presence of the DMSO alone or titrated concentrations of Deguelin as indicated for up to 3 days. Extents of cell proliferation judged by CFSE-dilution were analyzed daily by flow cytometry. Data obtained after treatments with other mitochondrial antagonists are also shown in Figure S3.

We first examined their effects on viability of resting primary T cells using ex vivo purified whole lymph node cells (Figure 6A). Treatments (24 hours) of the lymph node cells with the respective mitochondrial antagonists at concentrations near the IC_80_s for the pMV-absorption (Deguelin: 750 nM, Atpenin: 3000 nM, Antimycin: 12.5 nM, Oligomycin: 5 nM, FCCP: 2500 nM) resulted in sizeable decrease in viability of the T cells. Thus, only 20–33% of T cells survived, depending on the mitochondrial antagonist treated, while 67% of the T cells survived when they were treated with DMSO alone.

Noticeable increases in viability of the T cells were observed when the lymph node cells were treated at concentrations near the IC_50_s for the pMV-absorption (Deguelin: 300 nM, Piericidin: 2 nM, Atpenin: 1200 nM, Antimycin: 5 nM, Oligomycin: 2 nM, FCCP: 1000 nM). Nevertheless, viabilities of the drug-treated T cells were still lower than that of DMSO-treated cells; approximately 35–50% of the T cells survived after the culture, depending on the mitochondrial antagonist treated (Figure 6A). When the lymph node cells were treated at the concentrations 2–3 fold lower than the IC_50_s (Deguelin: 120 nM, Piericidin: 0.8 nM, Atpenin: 480 nM, Antimycin: 2 nM, Oligomycin: 0.8 nM, FCCP: 400 nM), viabilities of the T cells improved further to the level comparable to that of the DMSO-treated cells; viabilities of the drug-treated T cells, except for Atpenin and Oligomycin-treated cells, were close to or above 90% of that of the DMSO-treated cells (Figure 6A).

### Effects of Long-Term Mitochondrial Drug Treatments on Activation and Proliferation of Antigen-Stimulated T Cells

2C T cells undergo robust cell proliferation when they are cultured with nanometric membrane vesicles expressing cognate pMHC (i.e., L^d^/QL9 or L^d^/p2Ca) plus costimulatory ligands (i.e., B7-1 and ICAM-1); the cell division begins to occur after two days of culture and continues up to three days [13], [14]. We examined effects of the mitochondrial drug treatments on the activation (blast formation) and proliferation (CFSE dilution) of 2C T cells by QL9-loaded L^d^B7-1ICAM-1 pMVs (Figure 6B and C and Figure S3).

When the cells were cultured for 24 hours with the pMVs in the presence of the respective mitochondrial antagonists at concentrations near the IC_80_s for the pMV-absorption, increased population of 2C T cells died during the culture; approximately 35–45% of the drug-treated T cells survived depending on the mitochondrial drug used in the experiment while about 65% of the DMSO-treated cells survived. Average sizes of the surviving drug-treated 2C T cells were larger than that of 2C T cells cultured with the P1A-loaded pMVs but smaller than that of 2C T cells cultured with QL9-loaded pMVs in the presence of DMSO alone (Figure 6B and S3). The drug treatments completely inhibited the proliferation of 2C T cells; thus, the surviving drug-treated 2C T cells gradually died without cell division (CFSE dilution) as the culture time extended (Figure 6C and Figure S3). (Note: FCCP treatment, as shown in Figure S3, was the exception in that a population of surviving 2C T cells formed the blast to a greater extent and underwent several rounds of cell division.).

Significant improvements in cell viability and blast formation were found when 2C T cells were cultured for 24 hours with the respective mitochondrial antagonists at concentrations near the IC_50_s for the pMV-absorption (Figure 6B and Figure S3). The effects of the drug treatments were still apparent, however, so that only 50–55% of 2C T cells survived after the culture and the average sizes of the surviving cells were still considerably smaller than that of the DMSO-treated cells. Different from the 2C T cells surviving the 24 hour treatments at concentrations near the IC_80_s for the pMV absorption, a significant population of 2C T cells surviving the treatments at concentrations near the IC_50_s entered cell division cycle to proliferate. Nonetheless, the cell cycle progression was greatly impeded; thus the drug-treated cells underwent one cell division at most after 2 days of the culture while the DMSO-treated cells underwent up to three cell divisions. When the cells were examined after three days of the culture, the DMSO-treated cells underwent more than six cell divisions almost completely diluting out the CFSE while the drug-treated cells underwent less than four cell divisions (Figure 6C and Figure S3).

When 2C T cells were cultured with QL9-loaded L^d^B7-1ICAM-1 pMVs with the respective mitochondrial antagonists at concentrations 2–3 fold lower than the IC_50_s for the pMV-absorption, viabilities of 2C T cells increased further to the level comparable to that of the DMSO-treated cells (Figure 6B and C and Figure S3). In contrast, blast formation of 2C T cells was still significantly affected by the drug treatments, except for Antimycin and FCCP treatments. In parallel, proliferation of 2C T cells was also found to be impeded by those treatments. The effects of Deguelin and Atpenin were particularly noticeable; thus 2C T cells treated with each of those compounds underwent only 1 and 3–4 cell divisions after 2 and 3 days of the culture, respectively.

## Discussion

While 2C T cell absorption of L^d^B7-1ICAM-1 pMVs proceeds via complex mechanisms, the assay for measuring extents of the pMV-absorption is simple, expeditious and statistically accurate. In addition, considering that primary resting T cells isolated from lymph nodes of TCR Tg mice along with physiological ligands expressed on biological membrane are used, results obtained using the assay may hold superior physiological relevance. Those features make the assay system ideal for systematic investigation of cellular/molecular mechanisms underlying the pMV-absorption, namely structural basis for TCR/pMHC and LFA-1/ICAM-1 interactions and ‘inside-out’ signaling for TCR-mediated LFA-1 activation, via chemical genetics approach [15].

Applying the assay, we had established a cell-based flow cytometric HTS platform and screened a small molecule library to validate the system [15]. One of the intriguing findings from the pilot-scale HTS campaign was that various mitochondrial antagonists with distinct mechanisms of action ubiquitously inhibited the pMV-absorption without compromising the viability of 2C T cells (Figure 1). The acute inhibition of the pMV absorption by the mitochondrial antagonists suggested an unconventional role of mitochondria of regulating receptor-mediated membrane-proximal signaling cascades, e.g., TCR-mediated ‘inside-out’ signaling.

One may argue that the mitochondrial antagonists inhibit the pMV-absorption by depleting the pool of ATP and thereby suppressing activities of signaling molecules using ATP as a substrate, e.g., kinases. According to results from the HPLC analysis of 2C T cell extracts prepared after the mitochondrial drug treatments at concentrations around IC_80_s for the pMV-absorption (Figure 2A), the ratio of concentration of ATP to that of entire pool of adenine nucleotides decreased by a maximum of about 25%, which occurs when the cells were treated with Deguelin, and by an average of 15%. Changes in cellular energy balance were hardly detected when 2C T cells were treated with the drugs, except for Deguelin and Phenformin, at concentrations near the IC_50_s; even with Deguelin and Phenformin, decrease in the ratio of concentration of ATP to that of entire pool of adenine nucleotides was only about 4%. Given the marginal changes in the ATP ratio after the mitochondrial drug treatments, it seems unlikely that ATP depletion is a main reason for inhibition by the mitochondrial antagonists of the pMV-absorption.

Dose-dependent activation of AMPKα1 after the mitochondrial drug treatments was expected. Nonetheless, it is of note that significant levels of AMPKα1 activation were observed after treatments of 2C T cells with the respective mitochondrial drugs at concentrations near the IC_50_s where changes in cellular energy balance were almost undetectable (Figure 2A and B). With regard to that discrepancy, one may postulate that the cellular energy balance affected by the mitochondrial drug treatments was quickly restored to a homeostatic level following the AMPKα1 activation. That is, activated AMPKα1 suppressed ATP-consuming metabolic processes to balance the rate of ATP production and consumption and thereby the ratio among adenine nucleotides was maintained at the constant level [32]. Alternatively, it is also conceivable that the subtle change in cellular energy level hardly detected by UV absorption HPLC detector was able to induce phosphorylation of AMPKα1.

It is puzzling that mitochondrial antagonists with different modes of action have different effects on superoxide production (Figure 2C). It was, however, shown by studies using isolated mitochondria in vitro that the component of electron transport chain predominantly responsible for the production of SO varies depending on energy state of mitochondria. According to those studies, while complex III is the main complex for SO production in mitochondria of high respiratory activity (state III), complex I plays a major role in mitochondria of low respiratory activity (state IV). Results shown in Figure 2C could be understood in the context of those findings [10], [33], [34].

We examined the SO level to find whether changes in the SO level by the mitochondrial drug treatments could account for the ubiquitous inhibition of the pMV-absorption by those treatments. Given the differential effects, it seems difficult to explain the inhibition of the pMV-absorption with the perturbation of SO production by the drug treatments.

Phosphorylation of Akt and PLC-γ1 and entry of extracellular Ca^2+^, key signaling events for T cell activation, are preceded by activation of multiple upstream signaling molecules immediately following TCR triggering, which include PTK(s) and PI3K[35]. F-actin also plays a role in those signaling events. Dynamic F-actin rearrangement results in directional relocation of signaling molecules bringing them into close proximity and facilitating inter-molecular interactions among them [30]. In addition, costimulatory receptor/ligand interactions (e.g., CD28/B7-1 and LFA-1/ICAM-1) also make strong contribution to the phosphorylation of Akt and PLC-γ1 as well as entry of extracellular Ca^2+^ [26], [27], [28]. Thus, although results that the mitochondrial drug treatments inhibited the phosphorylation of Akt and PLC-γ1 and Ca^2+^ entry suggested a mitochondria-mediated control mechanism for membrane-proximal TCR signaling events (Figure 3), it was difficult to know whether the inhibition resulted from down-regulation of a specific signaling event or suppression of overall signaling process.

Here, results from the experiments for F-actin polymerization provide an important clue (Figure 4). Different from phosphorylation of Akt and PLC-γ1, F-actin polymerization in 2C T cells cultured with QL9-loaded L^d^B7-1ICAM-1 pMVs is solely dependent on 2C TCR/L^d^/QL9 interaction. Thus, extents of the F-actin polymerization are not affected by mAb blocking of either CD28/B7-1 or LFA-1/ICAM-1 interaction [15]. F-actin polymerization proceeds via a complex signaling process including activation of kinases (i.e., PTKs and PI3K), adapter proteins (i.e., Vav) and a WASP family protein (i.e., WAVE), etc [36], [37]. Together, results that the mitochondrial drug treatments had little effects on the F-actin polymerization (Figure 4) lead us to draw a conclusion that a branch of TCR signaling events progresses normally even after the mitochondrial drug treatments.

In agreement with the results from the flow cytometric analysis, microscopic analysis of 2C T cells cultured with the QL9-loaded pMVs showed enhanced cortical F-actin staining regardless of the mitochondrial drug treatments (Figure 5). Nevertheless, clear differences in distribution of cortical F-actin and in cell morphology were observed between DMSO- and mitochondrial drug-treated 2C T cells, indicating a process for dynamic rearrangement of F-actin was impaired by the drug treatments. Proper rearrangement of F-actin is the key for cell movement and directional relocation of signaling proteins [30], [38]. Dynamic rearrangement of F-actin proceeds via a series of signaling events involving kinases, phosphatases, adopter molecules and a host of actin binding proteins [39].

Taken together, we hypothesize that the mitochondrial drug treatments result in, by an unknown mechanism, inhibition of a specific signaling mechanism involved in dynamic rearrangement of F-actin but not in F-actin polymerization. We may understand the inhibition of the phosphorylation of Akt and PLC-γ1 in the same context. Thus, even though upstream signaling cascades commonly involved in phosphorylation of Akt and PLC-γ1 and F-actin polymerization (e.g., activation of PTKs and PI3K) operates normally after the mitochondrial drug treatments, the drug treatments attenuate F-actin rearrangement to compromise directional relocation of signaling molecules in close proximity to each other and thereby inhibit the activation of downstream signaling molecules such as Akt and PLC-γ1.

Importance of AMPK activation in inhibition by the mitochondrial antagonists of the pMV-absorption is yet to be resolved. Even though the strong inhibition by AICAR (5-aminoimidazole-4-carboxamide-1-β-riboside), a well-known AMPK activator [40], of the pMV-absorption by 2C T cells indicates the role of AMPKα1 in control of TCR signaling for LFA-1 activation (authors' unpublished data), that result has to be interpreted with caution. Studies by others have shown that AICAR treatment could have effects on certain metabolic processes independent of AMPK activation [41]. Studies using 2C T cells genetically deficient in AMPKα1 expression may help to understand the importance of AMPKα1 in regulation of TCR signaling and LFA-1 activation [42].

Signaling via TCR is crucial not only for activation and clonal expansion of T cells encountering foreign peptide/MHC complexes but also for survival of resting T cells. TCR signaling for the T cell survival is instigated as T cells encounter specific self peptide/MHC complexes that direct development (positive selection) of those T cell in thymus and are also presented by neighboring cells in secondary lymphoid organs (lymph nodes and spleen) [31]. The TCR/pMHC interactions are not strong enough to induce activation of downstream signaling events for cell cycle progression but sufficient to induce signaling events for maintaining integrity of cells. Given those, expected was the decrease in viability of resting lymph node T cells and 2C T cells cultured with the pMVs following longer treatments with the mitochondrial antagonists at the concentrations where the pMV absorption by 2C T cells was affected to measurable extents; i.e., concentrations near the IC_80_s and IC_50_s for the pMV absorption (Figure 6).

An important finding from the experiments shown in Figure 6 is, as manifested by treatments with Deguelin, that mitochondrial drug treatments at carefully controlled conditions may selectively inhibit activation and proliferation of antigen-stimulated T cells without compromising viability of neighboring resting T cells. While it is yet to be understood how the mitochondrial drug treatments slow the process of T cell activation and proliferation, the retardation of the blast formation by the drug treatments of 2C T cells cultured with the QL9-loaded pMVs appeared in line with the observation that the treatments interfered with dynamic reorganization of 2C T cells upon culture with the pMVs (Figure 5).

In order for certain compounds to be used as T cell immune modulators, it is desirable for them to show little toxicity against resting T cells in lymph nodes and spleen at the concentrations where they exert anti-proliferative effects on T cells undergoing activation process. In that regard, the results obtained with Deguelin are particularly encouraging. Here, studies having revealed cancer chemo-preventive effect of Deguelin are worth noting [43]. It is also of interest that chemo-preventive and anti-inflammatory effects of various oriental medicines and herbs are at least in part exerted by ingredients known to have an activity of antagonizing mitochondrial function [44], [45], [46].

## Materials and Methods

### Ethics Statement

All procedures met the guidelines of the National Institutes of Health “Guide for the Care and Use of Laboratory Animals”.

### Animals

C57BL/6J (B6) mice were purchased from The Jackson Laboratory (Bar Harbor, ME). 2C TCR Tg mice [47] were bred in the vivarium of The Scripps Research Institute (TSRI).

### Cell Lines and Culture Medium

Dros APCs were maintained in Schneider's medium supplemented with 10% fetal bovine serum (FBS) plus antibiotics and glutamine as described [48]. Short-term T cell cultures were performed in DMEM medium (Invitrogen) supplemented with 10% heat inactivated (HI) FBS plus antibiotics and glutamine. T cell proliferation assays were performed in RPMI medium supplemented with 10% HI FBS, 10 mM HEPES (pH 7.2), antibiotics, glutamine and 5×10^−5^ M 2-mercaptoethanol.

### Peptides, Chemicals and Antibodies

QL9 (QLSPFPFDL) and P1A (LPYLGWLVF) [48] peptides were purchased from Invitrogen.

Rotenone, Deguelin, Piericidin, Antimycin, Oligomycin, FCCP, Metformin and Phenformin were purchased from Sigma-Aldrich. Atpenin (A5) was purchased from Alexis Biochemicals (Farmingdale, NY). PP2 and Wortmannin were purchased from EMD Bioscience. Carboxyfluorescein succinimidyl ester (CFSE), Indo-1/AM, Dihydroethidium and Annexine V apoptosis detection kit were purchased from Invitrogen.

Anti-CD11a (M17/4), Alexa 488-conjugated Thy-1 (30-H12), PE-conjugated anti-B7-1 (16-10A1) and Alexa 647-conjugated anti-CD8 (53–6.7) were purchased from Biolegend (San Diego, CA). FITC-labeled Phalloidin was purchased from Sigma-Aldrich. Anti-CD8 (3.168) mAb was prepared as an ascites form (a gift from Dr. Surh in Department of Immunology at TSRI). Polyclonal antibodies directed to pan-Akt, phospho-Akt (Thr^308^), phospholipase C-γ1 (PLC-γ1), phospho-PLC-γ1 (Tyr^783^), AMPKα1, phospho-AMPKα1 (Thr^172^), Acetyl-CoA Carboxylase (ACC) and phospho-ACC (Ser^79^) were purchased from Cell Signaling Technology. Horse radish peroxidase (HRP)-tagged goat anti-rabbit IgG Ab was purchased from Santa Cruz Biotechnology.

### Preparation of Dros pMVs

PMVs were prepared from Dros APCs expressing L^d^ alone or L^d^ plus B7-1 and ICAM-1 as described previously [14], [15].

### Purification of 2C TCR Tg T Cells

2C T cells were negatively purified from pooled lymph nodes using CD8a^+^ T cell isolation MACS^TM^ kit (Miltenyi Biotech). If needed, the MACS purified 2C T cells were purified further by panning on anti-CD8 mAb (3.168)-coated plates to isolate CD8^+^ 2C T cells.

### Assay for 2C T Cell Absorption of L^d^B7-1ICAM-1 pMVs

The pMV-absorption assays were performed as described (15). Briefly, 45 µl of purified 2C T cells (4.5×10^5^) in a pre-warmed culture medium (DMEM medium) were mixed with 5 µl of the peptide-loaded L^d^B7-1ICAM-1 pMVs (0.5 mg/ml) in round-bottom 96 well plate and cultured for 30 min in a 37°C humidified CO_2_ incubator. After culture, PE-conjugated anti-B7-1 plus Alexa 647-conjugated anti-CD8 mAbs were added and the plate was incubated on ice for 20 min. The plate was then filled with ice-cold FACS buffer (1X PBS, 1% BSA, 2.5% horse serum, 2 mM EDTA) and spun down for cell washing. The T cells were resuspended in the FACS buffer containing propidium iodide (PI) and immediately analyzed by flow cytometry.

For compound testing, 2C T cells were incubated with 0.5 µl of respective compounds in DMSO for 60 min at 37°C prior to culture with the pMVs.

### Cell Viability Assays

Annexin V staining was performed following manufacturer's manual with purified 2C T cells treated with titrated concentrations of respective mitochondrial antagonists for 90 min.

Effects of long-term drug treatments on viability of T cells were examined as follows. Ex vivo purified whole body lymph node cells (B6) (1×10^6^) in 200 µl of RPMI medium were treated with titrated concentrations of respective mitochondrial antagonists for 24 hrs in a 37°C humidified CO_2_ incubator. The cells were then stained with Alexa 488-conjugated anti-Thy-1 mAb plus P.I. (5 mg/ml) and analyzed by flow cytometry.

### HPLC Analysis of Adenine Nucleotides

Purified 2C T cells (3×10^6^) in 1.5 ml of pre-warmed culture medium (DMEM medium) were treated with titrated concentrations of respective mitochondrial antagonists for 80 min at 37°C. Then, the cells were washed and lyzed in 250 µl of 10% ice-cold trichloroacetic acid. The cell lysates were spun down to remove cell debris and the supernatants were neutralized with Freon/n-octylamine mix (3∶1). The supernatant-Freon mixtures were spun down to separate the phases. Adenine nucleotides in the aqueous phase were then analyzed by HPLC using anion exchange column (Whatman Partisil 10-SAX column). After injection of 100 µl of sample, adenine nucleotides were eluted by linear gradient of 5 mM ammonium dihydrogen phosphate at pH 3.2 and 750 mM ammonium dihydrogen phosphate at pH 4.0 at a flow rate of 1 mL/min for 50 min [49].

### Flow Cytometric Analysis of Intracellular Superoxide (SO)

Purified 2C T cells (1×10^5^) in 100 µl of culture medium (DMEM) were treated with titrated concentrations of respective mitochondrial antagonists for 30 min at 37°C. Then, diethylethidum were added to the culture to 5 µM and incubated for 30 min at 37°C. The diethylethidium-treated 2C T cells were then stained with Alexa 647-conjugated anti-CD8 mAb for 10 min on ice, washed and resuspended in propidium iodide-containing FACS buffer (1X PBS, 10 mM HEPES, 2.5% horse serum, pH 7.2) and analyzed by flow cytometry.

### Flow Cytometric Analysis of F-Actin Content

Purified 2C T cells (2×10^5^) in 100 µl of culture medium (DMEM medium) were treated with titrated concentration of respective mitochondrial antagonists for 60 min before culture with L^d^B7-1ICAM-1 pMVs for 15 min. The 2C T cells were then fixed with paraformaldehyde (PFA) and stained with FITC-labeled Phalloidin and analyzed by flow cytometry as described previously [15].

### Confocal Microscopy

Purified 2C T cells (1×10^5^) in 100 µl of culture medium (DMEM medium) were treated with respective mitochondrial antagonists for 60 min before culture with L^d^B7-1ICAM-1 pMVs for 20 min on pre-warmed poly-L-lysine-coated coverslips. Then, the cells were fixed with PFA and stained with FITC-labeled Phalloidin and observed under the confocal microscope as described previously [15].

### Western Blotting Analysis

Preparation of cell extracts and Western blotting analysis were performed as described [15].

### Calcium Assay

Kinetic analysis of changes in cytosolic Ca^2+^ concentration was performed as described [50]. Briefly, 700 µl of purified CD8^+^ 2C T cells (1.4×10^6^) were loaded with 3 µM Indo-1/Am (Invitrogen) for 50 min at 37°C followed by thorough washing with ice-cold 1X PBS and resuspended in the culture medium (DMEM medium). Seven hundred microliters of Indo-1-loaded 2C T cells (1.5×10^6^) were treated with respective mitochondrial antagonists in DMSO (0.5% V/V) for 60 min in a 37°C water bath and mixed with 70 µl of peptide-loaded pMVs (1 mg/ml). Flow cytomeric analysis began 60 sec before addition of pMVs and continued for 720 sec afterward; FACS analysis was carried out with a LSR II flow cytometer equipped with a temperature-control device. Changes in the concentration of intracellular Ca^2+^ were determined measuring changes in the ratio of fluorescence intensities of Indo-1-loaded T cells detected by two separate photomultiplier tubes with bandpath filters of 440/40 (violet) and 530/30 (blue) nm, respectively; Indo-1 was excited by 351 nm UV laser.

### In Vitro T Cell Proliferation

CFSE-labeled purified CD8^+^ 2C T cells (5×10^4^) in 90 µl of culture medium (RPMI) were cultured with 10 µl of L^d^B7-1ICAM-1 Dros pMVs (0.5 mg/ml) loaded with either P1A or QL9 peptide in 96 well flat bottom plate in a 37°C humidified CO_2_ incubator. Extents of cell proliferation were determined by extents of CFSE dilution measured daily by flow cytometry [51]. At the beginning of the culture, 1 µl of respective mitochondrial antagonists in DMSO was added.

## Supporting Information

Figure S1 HPLC analysis for adenine nucleotides. Adenine nucleotides, marked by arrows (black: AMP, yellow: ADP, red: ATP), in cell extracts were separated and quantitated by HPLC. Chromatograms obtained with cell extracts of 2C T cells treated with either DMSO or Deguelin at 300 nM and 750 nM, respectively, are shown as examples.
(0.03 MB PDF)

Figure S2 Inhibition by the mitochondrial antagonists of activation of Akt and PLC-g1 by Ld pMVs X-ray films developed after WB analysis (Figure 3B) were scanned and intensities of each band relative to that of 2C T cells treated with DMSO alone and cultured with QL9-loaded Ld pMVs were calculated and plotted. Band intensity of each phospho-protein was compensated by that of the total protein in the corresponding sample.
(0.02 MB PDF)

Figure S3 Effects of long-term mitochondrial drug treatments on viability and activation of 2C T cells cultured with peptide-loaded LdB7-1ICAM-1 pMVs. Purified CFSE-labeled CD8+ 2C T cells were cultured with P1A- or QL9-loaded LdB7-1ICAM-1 pMVs in the presence of the respective mitochondrial antagonists or DMSO alone as indicated for up to 3 days. Extents of cell proliferation judged by CFSE-dilution were analyzed daily by flow cytometry. Percentage of live cells and their mean FSC measured after 1 day of the culture were denoted inside the histograms as in Figure 6B.
(0.11 MB PDF)
