# Supplementary figures and images for: Acute Inhibition of Selected Membrane-Proximal Mouse T Cell Receptor Signaling by Mitochondrial Antagonists
Source: PLoS One. 2009 Nov 10;4(11):e7738. doi: 10.1371/journal.pone.0007738 (PMC2768903; doi:10.1371/journal.pone.0007738)

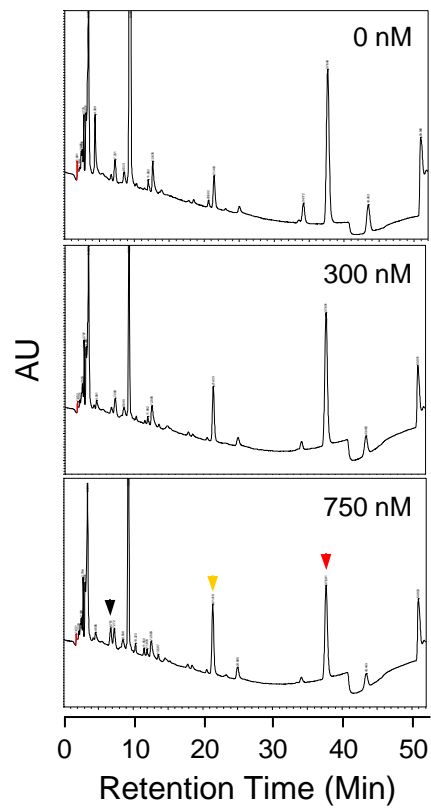

Supp. Fig. 1

Supplement: Figure S1 — HPLC analysis for adenine nucleotides. Adenine nucleotides, marked by arrows (black: AMP, yellow: ADP, red: ATP), in cell extracts were separated and quantitated by HPLC. Chromatograms obtained with cell extracts of 2C T cells treated with either DMSO or Deguelin at 300 nM and 750 nM, respectively, are shown as examples. (0.03 MB PDF) [file pone.0007738.s001.pdf]

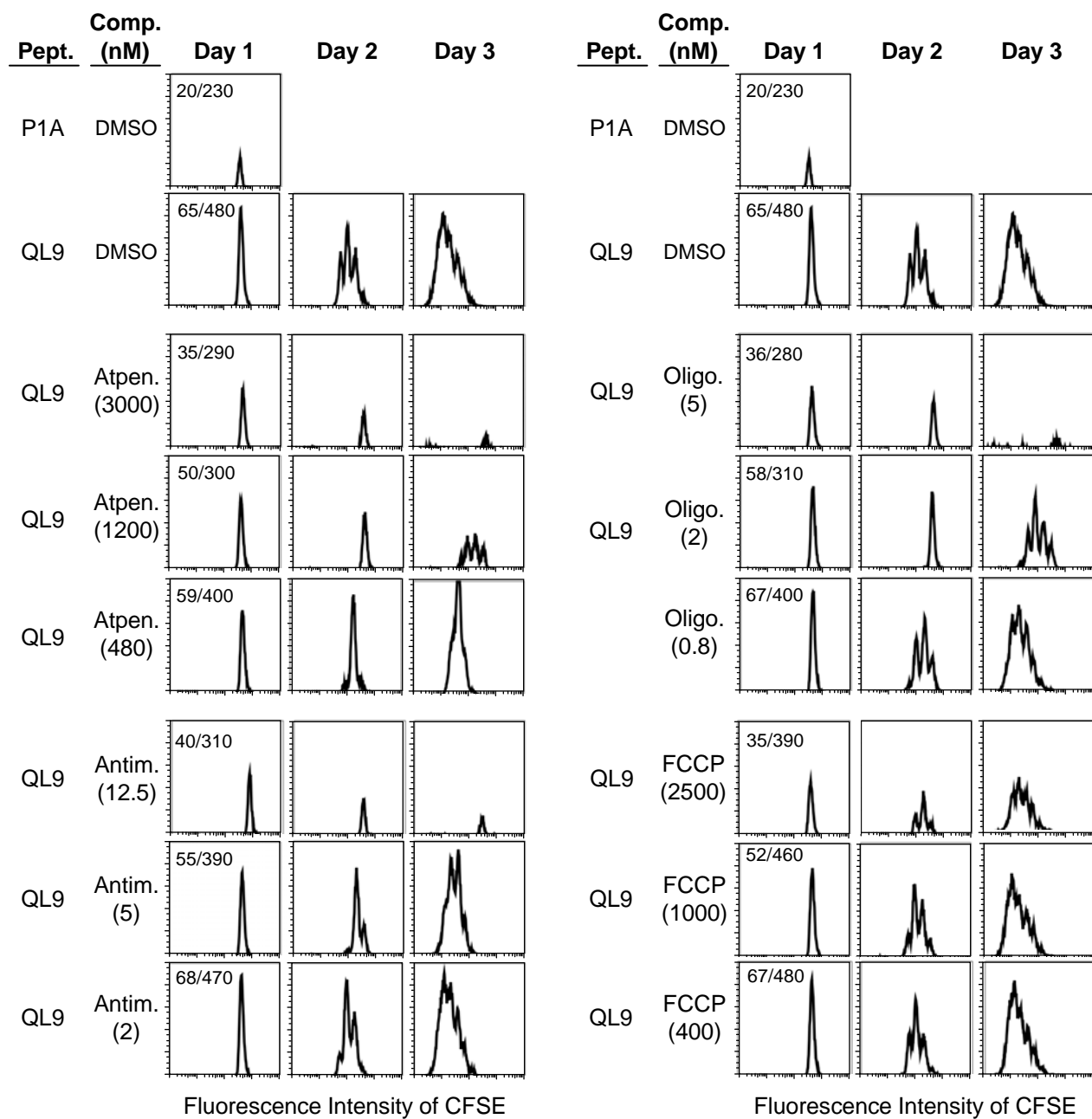

Supp Fig. 3

Supplement: Figure S3 — Effects of long-term mitochondrial drug treatments on viability and activation of 2C T cells cultured with peptide-loaded LdB7-1ICAM-1 pMVs. Purified CFSE-labeled CD8+ 2C T cells were cultured with P1A- or QL9-loaded LdB7-1ICAM-1 pMVs in the presence of the respective mitochondrial antagonists or DMSO alone as indicated for up to 3 days. Extents of cell proliferation judged by CFSE-dilution were analyzed daily by flow cytometry. Percentage of live cells and their mean FSC measured after 1 day of the culture were denoted inside the histograms as in Figure 6B. (0.11 MB PDF) [file pone.0007738.s003.pdf]
