# Supplementary material for: Acute Inhibition of Selected Membrane-Proximal Mouse T Cell Receptor Signaling by Mitochondrial Antagonists
Source: PLoS One. 2009 Nov 10;4(11):e7738. doi: 10.1371/journal.pone.0007738 (PMC2768903; doi:10.1371/journal.pone.0007738)
Supplement: Figure S2 — Inhibition by the mitochondrial antagonists of activation of Akt and PLC-g1 by Ld pMVs X-ray films developed after WB analysis (Figure 3B) were scanned and intensities of each band relative to that of 2C T cells treated with DMSO alone and cultured with QL9-loaded Ld pMVs were calculated and plotted. Band intensity of each phospho-protein was compensated by that of the total protein in the corresponding sample. (0.02 MB PDF) [file pone.0007738.s002.pdf]

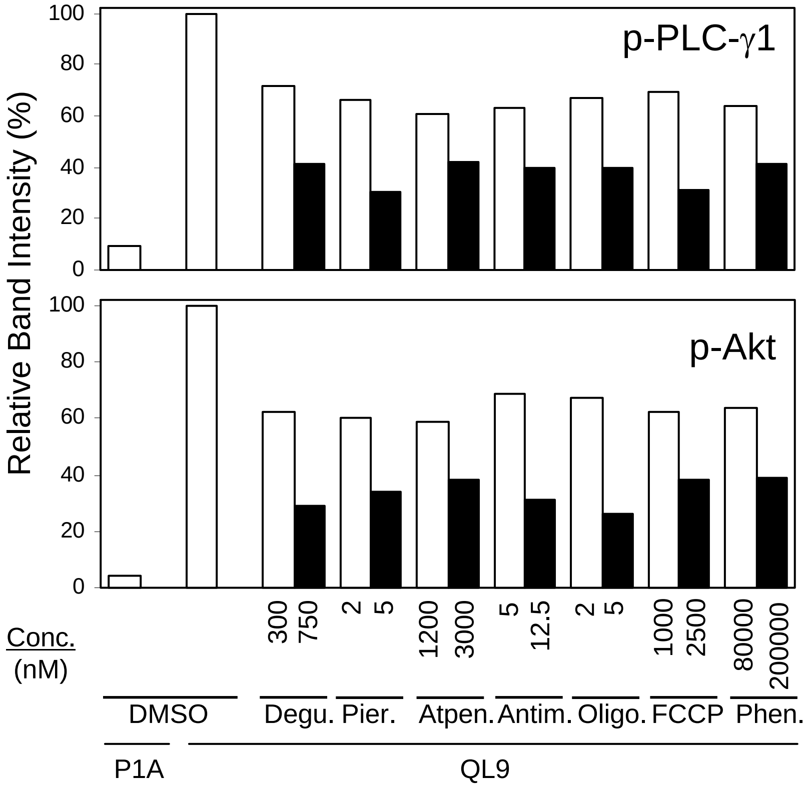

p-PLC-γ1
p-Akt
Relative Band Intensity (%)
100
80
60
40
20
0
Conc. (nM)
300
750
2
5
1200
3000
5
12.5
2
5
1000
2500
80000
200000
DMSO
Degu.
Pier.
Atpen.
Antim.
Oligo.
FCCP
Phen.
P1A
QL9

Supp Fig. 2
